# Medial Upper Lip Vermillion Reconstruction with a Labial Artery-based Cross-lip Vermillion Flap

**DOI:** 10.1097/GOX.0000000000002279

**Published:** 2019-06-25

**Authors:** Yoshihiro Sowa, Naoki Inafuku, Takuya Kodama, Daiki Morita, Toshiaki Numajiri

**Affiliations:** From the Departments of Plastic and Reconstructive Surgery, Kyoto Prefectural University of Medicine, Graduate School of Medical Sciences, Kamigyo-ku, Kyoto, Japan.

## Abstract

A defect of the central upper lip vermillion generally requires “like for like” reconstruction because this part of the upper lip can attract attention and has a unique structure and color. In this article, we report use of a labial artery-based horizontal long cross-lip flap for central upper lip vermillion reconstruction. In the first stage of surgery, a horizontal long vermillion flap from the lower lip starting at the left commissure with a vascular pedicle containing an inferior labial artery was raised and transposed to the upper vermillion defect. In the second stage, 12 days later, the pedicle was divided to complete the reconstruction. There were no postoperative complications in articulation or eating, and the patient was satisfied with the esthetic outcome. This surgical technique reduces microstomia and inconvenience in eating and speaking compared with a common horizontal cross-lip flap and provides better color- and texture-matched tissue compared to reconstruction using a tongue flap or mucosal flap. The technique is simple, requires a relatively short surgical time, has minimum donor-site morbidity and permits good esthetic and functional reconstruction of the central upper lip vermillion for a relatively small defect.

## INTRODUCTION

Upper lip reconstruction is still a surgical challenge due to the detailed lip anatomy, including the special texture of vermillion. “Like for like” reconstruction using an “Abbe” or “Estlander” flap for the upper lip has been widely performed. However, these flaps have the disadvantage of causing microstomia, and an Abbe flap causes inconvenience in eating and speaking because the flap bridging the upper and lower limits of mouth opening is free.^1,2^

Jin et al. introduced a 2-stage reconstruction procedure for a defect of the upper lip in patients with hemifacial atrophy, using a modified cross-lip vermillion flap with the pedicle specifically positioned at the commissure for functional and esthetic purposes.^3^ This technique reduces discomfort due to immobilization of the mouth for about 10–14 days before division of the pedicle, which resolves difficulties in speaking and eating and avoids compression of the pedicle of the flap intra- and postoperatively. We have used this flap for reconstruction of a central upper red lip following extensive resection of basal cell carcinoma. In this article, we describe the case of a patient with a central upper lip defect including the vermillion that was reconstructed using a horizontal flap with a labial artery combined with a full thickness skin graft. This procedure gave an ideal blend of cosmetic appearance and good blood supply.

## CASE REPORT

**Fig. 1.**
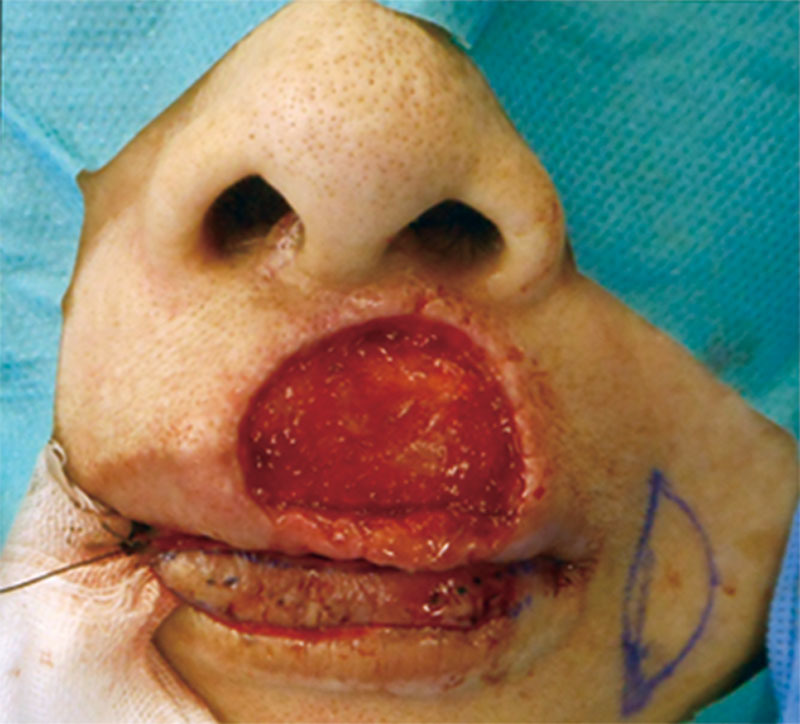
Preoperative frontal view showing the defect of the upper lip.

An 71-year-old woman presented with a 15 mm × 10 mm superficial basal cell carcinoma of the middle upper lip, which was excised with the cutaneous upper lip to vermillion, including part of the orbicularis oris. This resulted in a 23 mm × 18 mm defect over the upper lip that included the upper dry red lip and some of the wet red lip (Fig. 1).

## METHODS

The defect of the cutaneous upper lip and vermillion was reconstructed with a labial artery-based horizontal long cross-lip flap. Before the operation, the location of the inferior labial coronary artery was determined using a Doppler blood flow meter, and the flap was designed with correct inclusion of the artery. The anterior margin of the flap was located at the junction of the wet and dry vermillions. The vermilion flap containing the inferior labial coronary artery was elevated starting from the contralateral commissure. The surrounding orbicularis oris muscle cuff was included at the basement of the flap. A 5 mg bolus of indocyanine green (ICG) was injected through the peripheral intravenous line 90 seconds after flap elevation, and the vascularity of the flap was checked by ICG angiography using an infrared camera (Photodynamic Eye [PDE] neo; Hamamatsu Photonics K.K., Hamamatsu, Japan). The tip of the flap that showed the weakest ICG fluorescence was removed for safe transplantation.

**Fig. 2.**
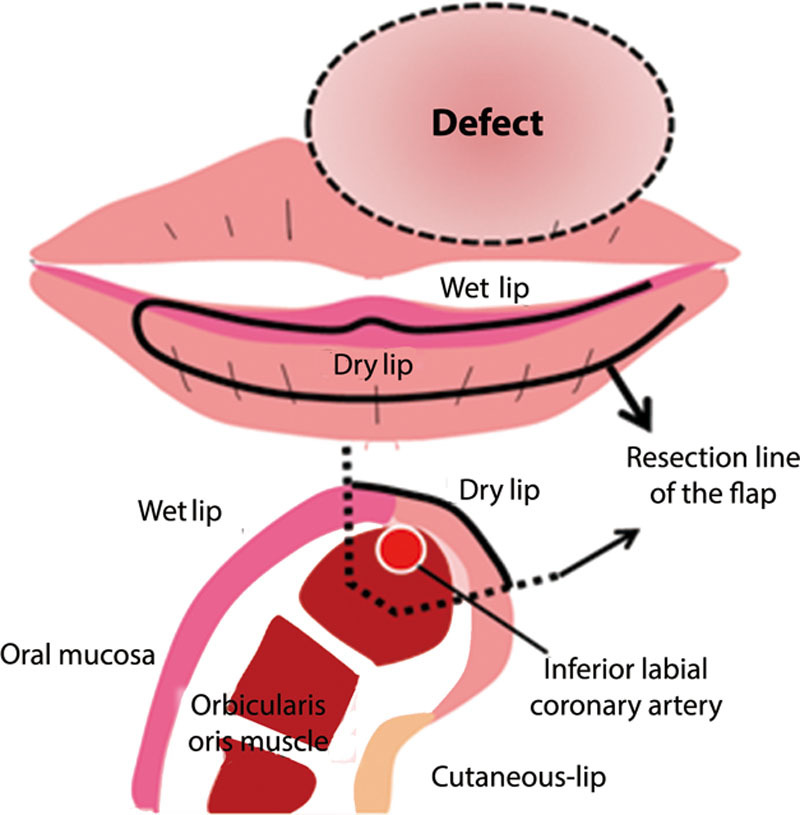
A schematic demonstration of the surgical design and procedures is shown.

**Fig. 3.**
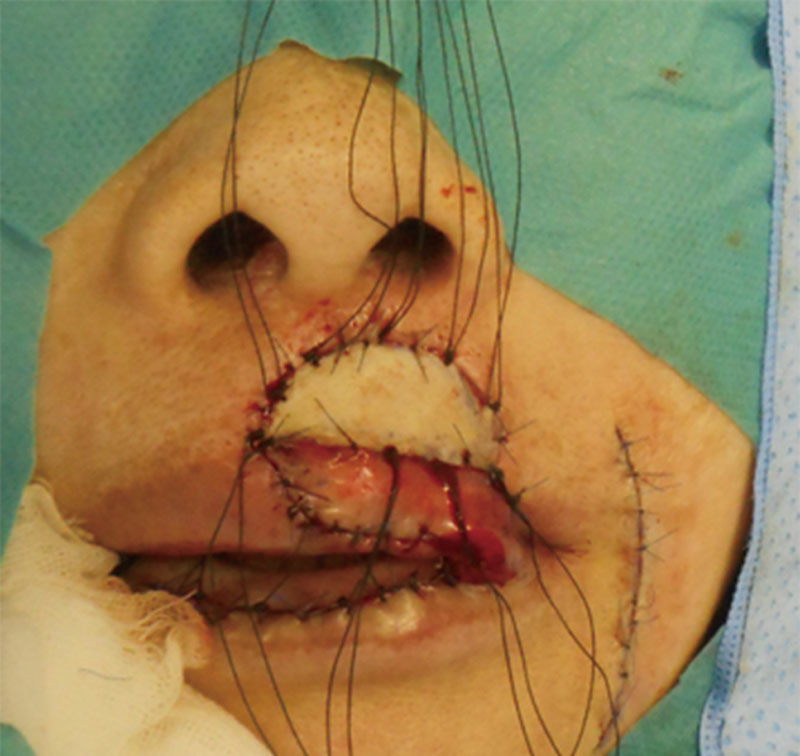
The flap was sutured into the defect of the upper lip vermillion and skin grafting was performed on the upper lip.

The maximum width of the harvested vermillion flap was 0.8 cm and the length was about 3.7 cm, giving a length:width ratio of approximately 4:1. In addition, the distal end of the flap was purposefully de-epithelialized to reconstruct the vermillion tubercle following satisfactory inset of the vermillion flap (Fig. 2). The defect of the cutaneous upper lip was reconstructed with full-thickness skin grafting from the left nasolabial fold (Fig. 3). The flap and dermis harvest sites were closed with direct suture. Nine days later, a delayed procedure was performed by clumping the base of the flap, and after another 3 days the pedicle was divided and redundant tissue was minimally trimmed to restore the appearance of the lateral commissure.

**Fig. 4.**
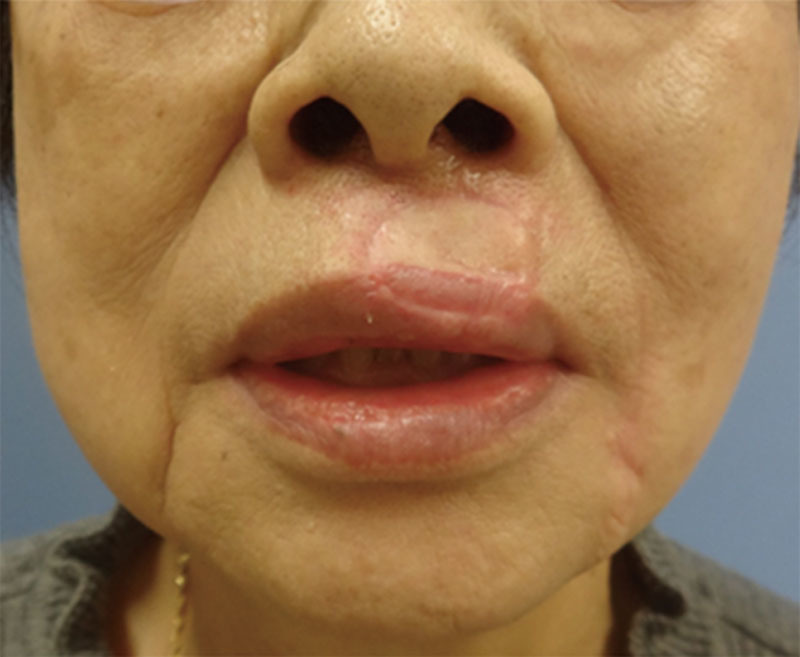
Postoperative view at 1 year.

The reconstructed upper vermillion was full and plump and largely symmetrical at 1 year after surgery (Fig. 4). The function of the oral sphincter and sensitivity of the red lip had fully recovered, and there had been no recurrence or metastasis of the tumor at the last checkup at 2 years after surgery. The morphology of the commissure was normal and the donor-site scar in the lower lip was inconspicuous. The patient remains satisfied with the surgical outcome.

## DISCUSSION

Vermillion reconstruction is sometimes required for a defect due to malignant tumor resection, trauma or congenital anomaly. Higher quality reconstruction is particularly required for a centrally located defect because this part of the upper lip easily attracts attention, even if the defect is not large. However, there are very few candidates as ideal tissue that is color- and texture-matched to the original tissue.

Use of normal vermillion tissues or adjacent tissues of similar color, such as an Abbe flap, tongue flap or buccal mucosal flap, can be used to achieve a reconstructed vermillion that is similar to the original.^1,2,4–6^ A vertical type cross-lip flap such as an Abbe flap is a good option for reconstruction of the central upper lip and can achieve ideal “like for like” reconstruction. However, this technique causes microstomia and requires a period in which the patient does not open his or her mouth because the pedicle is centrally located, which imposes an excessive burden on the patient.^7^ A tongue flap and buccal musculomucosal flap can be used to repair a large vermillion defect, but the color and texture of the tongue or oral vestibular mucosa do not completely match those of the vermillion.^4–6^ These techniques require application of lipstick to camouflage the color difference, and this make-up does not apply well to a newly reconstructed vermillion. Moreover, immobilization of the tongue before division of the pedicle is extremely uncomfortable for the patient and can cause difficulty in speaking and eating, as with the Abbe flap.

A labial artery-based horizontal long cross-lip flap resolves these unfavorable clinical problems and results in satisfactory esthetic and functional outcomes with minimum donor-site morbidity. This surgical technique is simple and not too time-consuming. However, care is required for successful and safe raising of the flap. One tip is that the labial coronary artery, which is a branch of the inferior labial artery extending from commissure to commissure, should be properly included in the flap pedicle. Preoperatively, this artery can be detected by ultrasound echo and marked, if possible including a vein. Second, it is recommended that ICG imaging is used for determining the extent to which the distal flap is vascularized. Third, if the lower lip is narrow in width, this technique is hard to use because collection of sufficient donor site tissue could reveal the oral mucosa, which loses the good appearance.

Our case required an additional donor site for cutaneous upper lip reconstruction with full-thickness skin grafting, but a scar is not evident if a nasolabial fold is used as the harvest site. In cases with deep defects through the full thickness of the vermillion, insufficient tissue volume may result in lip depression and whistle deformity. Therefore, our technique may be most appropriate for less than full-thickness defects of pure vermillion. When the full thickness of the vermillion including the cutaneous lip is resected, it may be better to select an Abbe flap for reconstruction because this method provides wide and bulky tissue.

To the best of our knowledge, this is the first report of use of a labial artery-based horizontal long cross-lip flap to reconstruct a unilateral upper lip vermillion defect after skin tumor resection. The surgical technique reported here provides a good esthetic and functional outcome for the central upper lip vermillion for a relatively small defect.
